# *In vivo* profiling of the PE/PPE proteins of *Mycobacterium tuberculosis* reveals diverse contributions to virulence

**DOI:** 10.3389/fmicb.2025.1634229

**Published:** 2025-06-18

**Authors:** Benjamin Koleske, Jessica Shen, Manish Gupta, William R. Bishai

**Affiliations:** Center for Tuberculosis Research, Department of Medicine, Johns Hopkins University School of Medicine, Baltimore, MD, United States

**Keywords:** *Mycobacterium tuberculosis*, transposon sequencing, PE/PPE proteins, virulence factors, bacterial secretion, type VII secretion systems

## Abstract

*Mycobacterium tuberculosis* (*M.tb*) uses a plethora of cell surface and secreted virulence factors to survive within the host. Among these are the PE/PPE proteins, a pair of secretory families that have expanded to 168 members in *M.tb*. Most of these proteins are poorly characterized due in part to their repetitive sequences and high similarity to one another. While *PE/PPE* genes are generally non-essential *in vitro*, many are highly expressed during animal infection. Thus, we conducted an *in vivo* pooled screen of 87 transposon mutants in *M.tb PE/PPE* genes and used Tn-seq to identify mutants with fitness defects in the mouse lung environment. We found consistent, time-dependent changes in mutant abundance across our animal replicates and identified decreases in several key mutant strains known to promote bacterial growth or virulence. In all, 27 of the 87 mutants showed significant reductions in percent population prevalence in the lung over 3 weeks. We then selected a transposon mutant in the *PPE71* gene and validated that this strain was attenuated in a single-strain infection. Our findings suggest that a high proportion of *PE/PPE* genes (31%) are required for virulence in the mouse model. These observations suggest that individual *PE/PPE* genes have differing contributions to virulence and may help prioritize future studies of these families. Strikingly, these properties were seen only in an *in vivo* model, which may imply a role for PE/PPE proteins in *M.tb* host-pathogen interactions.

## Introduction

Unlike other major bacterial causes of pulmonary disease (Carroll and Adams, 2016), *Mycobacterium tuberculosis* (*M.tb*) is not a common colonizer of the upper airway (Jindal et al., 2016) and lacks a known environmental reservoir (Gagneux, 2018). Instead, *M.tb* mainly resides in the human lung, where it blocks phagolysosome maturation of infected macrophages (Clemens and Horwitz, 1995) to render the intracellular environment more hospitable. This ability to disrupt host membranous compartments is primarily mediated by ESX-1 (Pym et al., 2002; Hsu et al., 2003; Gao et al., 2004), one of five specialized type VII secretion systems in *M.tb*. With the phagosomal membrane compromised, *M.tb* can export material into the cytoplasm of the infected phagocyte and directly extravasate out of the damaged phagosome (van der Wel et al., 2007; Simeone et al., 2012; Houben et al., 2012). Via these processes, the pathogen uses cell surface and secreted virulence factors to recruit naïve macrophages for infection (Cambier et al., 2014), disrupt autophagy to prevent bacterial eradication (Deretic, 2014; Strong and Lee, 2020), and promote necrotic cell death that aids bacterial replication (Ramakrishnan, 2012; Lerner et al., 2017). Hence, *M.tb* remodels host immune cells and tissue structures to establish a microenvironment, the granuloma, that permits bacterial survival in the host (Ramakrishnan, 2012; Flynn et al., 2011).

As mycobacteria have evolved, they have undergone four successive duplications of their type VII secretion systems (Cole et al., 1998; Gey van Pittius et al., 2006). ESX-5, the product of the most recent duplication, arose as mycobacteria were transitioning from a fast-growing lifestyle to the slow-growing behavior seen in *M.tb* and related pathogenic mycobacteria (Gey van Pittius et al., 2006). *M.tb* ESX-5 is necessary for virulence, as full or partial deletions of this secretory system cause attenuation (Bottai et al., 2012; Tiwari et al., 2020) or loss of viability (Di Luca et al., 2012; Ates et al., 2015). Unlike the other ESX systems, which secrete a small number of proteins, the ESX-5 system has over 150 confirmed and putative substrates dispersed all throughout the *M.tb* genome (Cole et al., 1998; Gey van Pittius et al., 2006; Ates et al., 2015; Kapopoulou et al., 2011; Abdallah et al., 2009). Most of these substrates belong to the PE and PPE families of proteins, named for the conserved Pro-Glu and Pro-Pro-Glu amino acids in their N-terminal secretion domains (Cole et al., 1998). While *PE/PPE* gene homologs are sporadically seen in other Actinobacteria with type VII secretion systems (McGuire et al., 2012), repeated duplication of these substrate proteins is characteristic of mycobacteria (Gey van Pittius et al., 2006). Furthermore, pathogenic mycobacteria have acquired unique subtypes of PE/PPE proteins, including the PE_PGRS (polymorphic GC-rich repeat sequence) and PPE-MPTR (major polymorphic tandem repeat) families, each named for their large repetitive domains (Gey van Pittius et al., 2006). While considerable work has been done on PE/PPE proteins, including the repetitive subfamilies, the function of many of these proteins remains unknown (Ates, 2020).

This knowledge gap surrounding the PE/PPE proteins can be explained in part by their unusual properties. The high similarity between PE/PPE family members and the relative rarity of basic residues for trypsin cleavage has rendered these proteins challenging to disambiguate by mass spectrometry (Ates, 2020; Schubert et al., 2013). Analogously, the extraordinarily high GC-content of many of these genes make them difficult to accurately sequence, and the repetitive domains further complicate efforts at genome assembly (Cole et al., 1998; Poulet and Cole, 1995; Modlin et al., 2021). Despite these complexities, work on individual PE/PPE proteins has yielded a range of intriguing proposed functions, including nutrient uptake (Tufariello et al., 2016; Wang et al., 2020; Boradia et al., 2022), protein export (Ates et al., 2018a), lipase activity (Mishra et al., 2008; Singh et al., 2016), and host-interacting processes (Ates, 2020; Sampson, 2011). This diversity defies the historical view of the PE/PPE proteins as mere sources of antigenic variation (Banu et al., 2002) and establishes them as a vast repertoire of functional capacity for *M.tb* (Copin et al., 2014). Yet, most *PE/PPE* genes have been found to be non-essential for *in vitro* growth (Sassetti et al., 2003; DeJesus et al., 2017), suggesting that their functions involve survival in the host. Indeed, PE/PPE proteins have been found to be among the most abundant *M.tb* proteins in lung tissue from infected animals (Kruh et al., 2010). The known localization of PE/PPE proteins to the extracellular environment (Ates et al., 2018a) or the outer mycomembrane compartment (Palande et al., 2024) suggest that these proteins would be exposed to the host during infection. As such, numerous studies have suggested that the PE/PPE proteins possess a role in modulating the host immune response during infection, whether as a primary function or a secondary moonlighting property (Tiwari et al., 2020; Ates, 2020; Sampson, 2011; Copin et al., 2014; Ates et al., 2018b).

To investigate a role for the PE/PPE proteins *in vivo*, we gathered single transposon mutants in 87 *PE/PPE* genes (approximately half of the *M.tb* repertoire), which had been isolated in previous studies by our group (Lamichhane et al., 2003; Lamichhane et al., 2005). Using a pooled design, we infected these mutants into mice by aerosol route and used transposon sequencing (Tn-seq) to measure changes over time in the proportional prevalence of each bacterial strain within the lung. We observed a strong correlation in the behavior of the strains at Week 1 and Week 3, indicating a time-dependent directional change in pool composition. For further study, we selected a transposon mutant in *PPE71* that declined in population prevalence over time in the pooled infection, suggestive of a virulence defect. We subsequently confirmed that this *PPE71*::Tn mutant was attenuated for animal infection by lung bacterial burden, lung histopathology, and animal survival. Overall, we observed consistent differences in the behavior of our many *PE/PPE* gene mutants *in vivo*, suggesting a highly varied contribution of individual family members to *M.tb* virulence. These changes were best elicited by an *in vivo* infection model compared to a macrophage infection model, suggesting the requirement of some aspect of host biology that is not replicated in a simpler cellular assay. Hence, our findings demonstrate the importance of studying the PE/PPE proteins in a full host immune context and suggest that this complexity may be needed to understand their functions.

## Materials and methods

### Bacterial culture

For growth of *M.tb* in broth, Middlebrook 7H9 was supplemented with 0.5% glycerol, 10% OADC, and 0.05% Tween 80 (henceforth ‘complete 7H9’). Cultures were grown at 37°C with shaking at 180–200 rpm. Growth curves were conducted by seeding from mid-log phase cultures to the equivalent of starting OD_600_ 0.006 in 10 mL. For growth of *M.tb* on solid agar, Middlebrook 7H11 was supplemented with 0.5% glycerol and 10% OADC. Plates were grown at 37°C for 3 weeks. Selection was achieved using 25 ug/mL kanamycin and/or 50 ug/mL hygromycin as needed.

For plasmid cloning, *Escherichia coli* strain DH5α was grown in LB broth or on LB agar at 37°C. Selection was achieved using 100 ug/mL carbenicillin or 50 ug/mL kanamycin as needed.

### *M.tb* transposon mutants

The *M.tb* transposon mutants used in this study were generated as part of previous work in which single transposon mutants were picked from individual colonies and sequenced (Lamichhane et al., 2003; Lamichhane et al., 2005). Each transposon mutant strain contains a single integration event of the *Himar1* transposon, which inserts into TA dinucleotides (Lampe et al., 1996) across diverse organisms, including mycobacteria (Rubin et al., 1999). The *Himar1* sequence acts as a well-defined mark for the site of insertion and contains a kanamycin resistance cassette to facilitate selection. Because the original work that developed these mutants used an *M.tb* CDC1551 background (Lamichhane et al., 2003; Lamichhane et al., 2005), we likewise selected *M.tb* CDC1551 (henceforth, ‘WT’) as our WT comparison. We were able to obtain 87 *M.tb* transposon mutant strains annotated as each having a transposon insertion in a distinct *PE/PPE* gene. If multiple transposon mutants were cataloged for a particular gene, we opted to use the strain with the most N-terminal insertion. For example, the *PPE71*::Tn strain (henceforth ‘71::Tn’) his its point of insertion at nucleotide 52 of 1,155 in the *PPE71* gene (MT2422). Additional details regarding the mutant strains can be provided upon request.

To generate the transposon mutant pool for mouse infection, 100 uL of each frozen stock was seeded separately into an individual 10 mL culture under kanamycin selection. Individual strains were frozen from each primary culture at OD 0.6 as they resuscitated; initial resuscitation time varied from 1 to 4 weeks. Subsequently, 50 uL of each individual secondary stock was then thawed into a fresh 4 mL culture. Compared to the initial resuscitation, growth of the secondary cultures was highly similar. Cultures were normalized to OD 0.5 by dilution, then 200 uL of each culture was pooled together, and 10 mL of this pool was used for mouse infection as described below.

### *M.tb* strain construction

To construct the *PPE71* complement, plasmid pMH94-hyg (Lee et al., 1991) was digested with XbaI. A fragment including the *PPE71* gene and its native promoter and UTRs was amplified from *M.tb* CDC1551 genomic DNA using PPE71_24f/r. This fragment was incorporated into the linearized plasmid by Gibson assembly to produce pMH94-hyg-PPE71. Sequences were verified using pMH94_Fseq/Rseq, and pMH94-hyg-PPE71 was transformed into the 71::Tn strain by electroporation.

To construct the *PPE71* overexpressor, plasmid pSD5 (DasGupta et al., 1998) was digested with NdeI and MluI. A fragment including the *PPE71* gene with minimal flanking sequences was amplified *M.tb* CDC1551 genomic DNA using PPE71_pSD5_f/r. This fragment was incorporated into the linearized plasmid by Gibson assembly to produce pSD5-71OE. Sequences were verified using pSD5_insert_FWD/REV, and pSD5-71OE was transformed into the WT strain by electroporation.

Electroporation of plasmid DNA into *M.tb* and extraction of *M.tb* DNA have been described previously (Larsen et al., 2007). Supplementary Tables S2, S3 summarize plasmids and oligonucleotides used in this work, respectively.

### RNA isolation and qRT-PCR

Isolation of total RNA from *M.tb* was performed as described previously (Bullen et al., 2023). Briefly, *M.tb* strains grown in 10 mL cultures to mid-log OD were pelleted, and pellets were resuspended in 1 mL TRIzol (Thermo). Bacteria were lysed by bead beating in a Precellys Evolution homogenizer for 3 cycles of 7,400 bpm for 30 s and kept on ice for 1 min between cycles. To each soluble fraction, 200 uL of chloroform was added, and the aqueous phase was obtained by phenol-chloroform extraction. RNA samples were purified using an RNeasy kit (Qiagen), per manufacturer’s instructions, using two incubations with DNase I (Qiagen) to remove residual bacterial chromosomal DNA. RNA concentration and purity was measured by 260/280 ratio on a NanoDrop spectrophotometer (Thermo).

*M.tb* RNA was converted to complementary DNA (cDNA) using qScript cDNA SuperMix (Quantabio), per manufacturer instructions. Real-time quantitative PCR (qRT-PCR) was performed using iTaq Universal SYBR Green Supermix (Bio-Rad), per manufacturer instructions, on a QuantStudio 3 Real-Time PCR System (Applied Biosystems) using a ∆∆Ct method. *M.tb* 16S rRNA was used as an internal normalization control for all transcripts. Sequences for primer pairs to amplify the *PPE71* transposon junction (PPE71_q_T1F/R), the 3’ UTR of *PPE71* (PPE71UTR_qF/R), the *esxX* gene (EsxX_qF/R), the 3’ UTR of *PPE38* (PPE38UTR_qF/R), and 16S rRNA (16S_q_F/R) are provided in Supplementary Table S3.

### Macrophage infection

To examine the behavior of the *PPE71* variant strains in macrophages across various timepoints, we used the J774 mouse macrophage-like cell line. J774 cells were cultured in Dulbecco’s modified Eagle’s medium (DMEM) (Gibco) supplemented with 10% fetal bovine serum (FBS) (Gibco). For infection, 0.2 million J774 cells per well in 24-well plates were infected with WT, 71::Tn, and 71comp strains at an MOI of 5 (1 million bacteria per well). Bacteria were allowed to infect the cells for 4 h, after which the cells were incubated with 100 ug/mL gentamicin for 1 h to kill remaining extracellular bacteria. Cells were washed into fresh medium, and 3 wells infected with each strain were harvested for the Day 0 timepoint by removing the culture media and applying 500 uL of 0.025% SDS in PBS for 10 min at room temperature. Bacterial CFUs were enumerated by preparing 10-fold dilutions into PBS and spreading these onto 7H11 plates. Cells were harvested at Day 1, Day 3, and Day 5 timepoints analogously. Culture medium was changed at Day 1 and Day 3 for surviving timepoints. Bacterial CFUs were counted after 3 weeks of incubation at 37°C.

To study the behavior of a variety of *PE/PPE* gene mutants in macrophages, we used mouse bone marrow-derived macrophages (BMDMs). Briefly, 5 female C57BL/6 J mice (#000664) were purchased from The Jackson Laboratory. Mice were humanely euthanized, and bone marrow was harvested from the femur and tibia bones. Marrow was filtered through a 70 um filter, and cells were washed into Roswell Park Memorial Institute 1,640 (RPMI 1640) medium containing GlutaMAX supplement (Gibco) supplemented with 10% FBS and 10% filtered supernatant from L929 feeder cells. Cells were washed into fresh medium after 4 days, then once again after another 3 days. One day prior to infection, mouse interferon gamma was added at a final concentration of 100 ng/uL to prime the BMDMs. For infection, 0.5 million BMDMs per well in 24-well plates were individually infected with 22 transposon mutant strains (see Supplementary Figure S2) and WT *M.tb* at an MOI of 2 (1 million bacteria per well). Following infection, cells were treated with gentamicin and washed as described above. At Day 0 and Day 2 timepoints, 2 wells infected with each strain were harvested using SDS and plated onto 7H11 as described above.

### Mouse infection and endpoints

For each animal study, 8-week-old female BALB/cJ mice (#000651) were purchased from The Jackson Laboratory. Mice were housed within an animal biosafety level 3 facility and provided *ad libitum* rodent chow and clean water. The facility was maintained on alternating 12-h light/dark cycles. Mice were infected by aerosol route using a Glas-Col Inhalation Exposure System.

For the pooled transposon mutant study, the live pool of *M.tb* mutants described above was implanted to yield an inoculum of approximately 1,500 CFUs per animal. A total of 10 mice each were sacrificed on Day 1, Week 1, and Week 3 post-infection. Combined left and right lungs were homogenized using Dounce homogenizers, and the total homogenate was distributed across 5, 10, or 20 7H11 plates for the Day 1, Week 1, and Week 3 timepoints, respectively. Plates were allowed to incubate for 3 weeks at 37°C, at which point bacterial genomic DNA was harvested from 5,000–10,000 colonies and used to prepare Tn-seq libraries, described below.

For the individual *PPE71* strain study, *M.tb* WT, 71::Tn, 71comp, and 71OE were individually infected into mice from frozen stocks to yield inocula of approximately 200 CFUs per animal. At Day 1, 3–4 animals per group were sacrificed to measure inocula by plating homogenate from the combined left and right lungs. At Week 4 and Week 8, 5 animals per group were sacrificed. Right lungs were homogenized using Dounce homogenizers and applied to 7H11 plates as a 10-fold dilution series. Plates were allowed to incubate for 3 weeks at 37°C, then bacterial CFUs were counted. Left lungs were fixed in 10% neutral-buffered formalin (NBF) for 2 days followed by incubation for 1 day in PBS. Fixed lungs were embedded, sectioned at 4 um, stained with hematoxylin and eosin (H&E), and imaged by brightfield microscopy. H&E images were manually processed in ImageJ to identify the proportion of each lung area occupied by lesions. For the survival study, 10 animals in each group were housed until reaching a natural death endpoint, consistent with our animal use protocol. Body weights were measured weekly for half of the animals (5 per group) until the first endpoint was noted.

### Tn-seq

To obtain genomic DNA for transposon sequencing (Tn-seq), *M.tb* colonies were scraped from 7H11 plates into a solution of 50 mM glucose, 25 mM Tris–HCl (pH 8.0), and 10 mM EDTA, as described in Larsen et al. (2007). All plates associated with a particular animal were pooled together, yielding 30 total samples (10 animals per each of 3 timepoints). To 10 mL of bacterial suspension, 500 uL of 10 mg/mL lysozyme was added, and samples were incubated at 37°C overnight. Genomic DNA extraction was performed as previously described (Larsen et al., 2007). DNA concentration and purity was measured by 260/280 ratio on a NanoDrop spectrophotometer (Thermo). To achieve fragments around 500 bp, 5 ug of DNA from each sample was sheared using a Qsonica Q125 sonicator on ice using the following settings: 10 min ON time, 60% amplitude, 10 s ON/10 s OFF.

To prepare libraries for next-generation sequencing, we adapted a previously described protocol for *M.tb* Tn-seq (Long et al., 2015). Briefly, sheared DNA was treated with an End-It DNA End-Repair kit (Lucigen) then A-tailed using dATP and Taq polymerase (New England Biolabs). Adapter_constant and Adapter_variable oligonucleotides were mixed at equimolar ratio and annealed in a thermocycler by slow ramp down from 95°C. Annealed adapters were ligated to each DNA prep using T4 DNA ligase (New England Biolabs). Transposon junctions were PCR amplified in a two-step process. For the first step, the Short_adapter/Short_transposon primer pair was used to perform four independent PCR reactions per sample, which were then pooled. Next, a hemi-nested PCR was performed using a mixture of four constant primers against the transposon (HN_transposon_1–4) and a mixture of four primers with a unique library-specific barcode (HN_barcode_XX-1–4, in which the ‘X’ positions represent one of 30 barcodes).

Libraries were pooled by equal mass into a single multiplexed pool. The pool was sequenced at a depth of 300 million 150 bp paired-end reads (approximately 10 million reads per sample) on an Illumina NovaSeq 6,000 using a NovaSeq SP flow cell (300 cycles). Adapter sequences were trimmed, and reads were bucketed by library barcode. To quantitatively map the sequencing data to the *M.tb* genome, the TRANSIT tool and the accompanying TRANSIT pre-processor (TPP) developed for analysis of *Himar1* Tn-seq data were used (DeJesus et al., 2015). TRANSIT outputted a list of counts at each TA site in the target genome; we then manually identified these locations in the *M.tb* genome to assign genes to the respective sites. Raw read counts were normalized to total reads for each animal.

Two libraries were ultimately excluded for logistical reasons. The 7H11 plates from mouse #8 (Day 1) were lost due to contamination with a non-*M.tb* organism, and so the genomic DNA obtained was poor quality. The sequencing of library #15 (Week 1) produced very few (<1,000) reads that could be assigned to a TA site, so it was omitted from further analysis.

Sequences of adapters and primers are provided in Supplementary Table S3. Barcode sequences, which replace the ‘X’ bases in the HN_barcode_XX primers, are provided in Supplementary Table S4. Barcode sequences 1–8 were adapted from prior work (Long et al., 2015), while the remaining barcode sequences were generated using the DNABarcodes package in BioConductor (Buschmann and Bystrykh, 2013) to generate a list of 8-mer nucleotide sequences resistant to indels and up to one miscode.

### Statistical analysis

Statistical analysis was performed in GraphPad Prism Version 10. Groups were analyzed for significant findings using one-way analysis of variance (ANOVA) tests followed by Tukey’s test for multiple comparisons. For survival analysis, a Mantel-Cox log-rank test was used to assess significance. Error bars in all figures represent mean ± standard deviation. All raw points are shown, and no points were omitted. All data points represent distinct samples; samples were not measured repeatedly. *R*^2^ values are provided for all linear regressions. In all cases, *p* < 0.05 or a more stringent threshold was used for statistical significance.

### Ethics statement

Experimental procedures were conducted in accordance with protocol #MO22M466 (formerly #MO20M20), approved by the Institutional Animal Care and Use Committee (IACUC) of Johns Hopkins University. Animal procedures were designed to eliminate or minimize animal distress and pathology.

## Results

### An *in vivo* Tn-seq design for studying *M.tb PE/PPE* gene mutants

**Figure 1:**
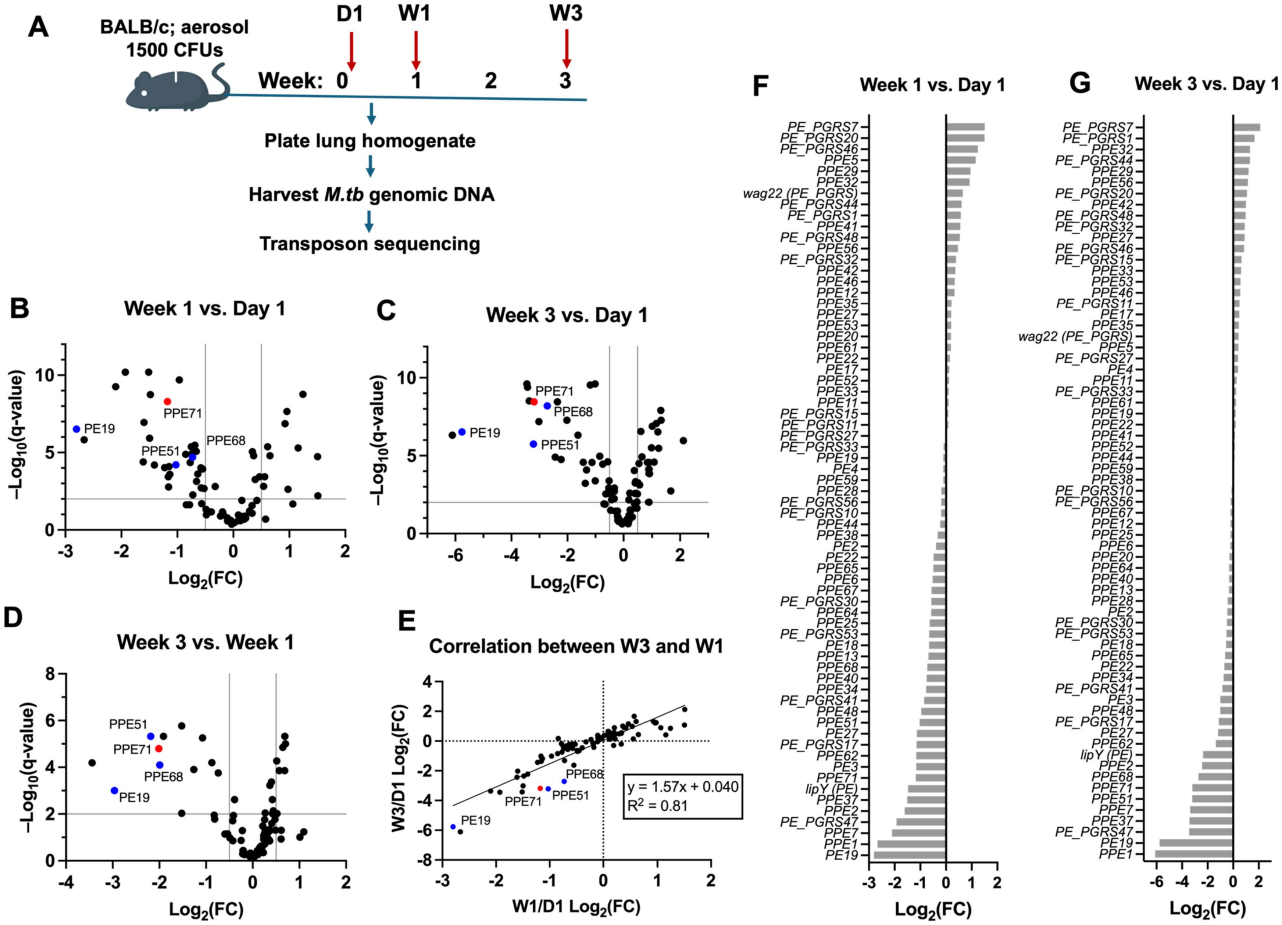
The *PE/PPE* families have a high abundance of genes required for virulence. **(A)** Design for high-dose pooled aerosol infection of *M.tb* transposon mutants into BALB/c mice. Animals were sacrificed on Day 1, Week 1, and Week 3 as experimental timepoints (*n* = 9–10 each). Lung homogenates were plated on 7H11 to culture *M.tb*, then genomic DNA was extracted from the bacteria was subjected to transposon sequencing. **(B–D)** Volcano plots depicting fold change in the population prevalence of each strain over time. Comparisons are shown for Week 1 vs. Day 1 **(B)**, Week 3 vs. Day 1 **(C)**, and Week 3 vs. Week 1 **(D)**. Indicated thresholds are ± 0.5 log_2_ (fold-change) (vertical lines) and *q* < 0.01 (horizontal line). Transposon mutants in *PE19*, *PPE51*, and *PPE68*, which are ‘positive controls’ known to promote *M.tb* virulence, are indicated in blue, while the mutant in *PPE71* is indicated in red. **(E)** Correlation plot comparing the proportional abundance of each bacterial strain at Week 3 and Week 1 timepoints (each normalized to Day 1 abundance by log_2_ (fold-change)). The strong positive correlation suggests consistent behavior of the mutants over time. **(F,G)** Log_2_ (fold-change) values for each *PE/PPE* gene mutant strain at Week 1 **(F)** and Week 3 **(G)**, each versus Day 1. Note that *lipY* is a PE-family gene, while *wag22* is a PE_PGRS-family gene.

The *M.tb* genome contains 97 *PE* genes and 71 *PPE* genes for a total of 168 *PE/PPE* genes (Cole et al., 1998). From a previously described collection of *M.tb* CDC1551 single gene *Himar1* transposon mutants (Lamichhane et al., 2003; Lamichhane et al., 2005), we resuscitated 87 strains, each annotated as having a transposon insertion in a unique *PE/PPE* gene. We grew these strains as separate cultures *in vitro*, normalized them by optical density at 600 nm (OD_600_), and pooled them together for aerosol infection into BALB/c mice. We selected Day 1, Week 1, and Week 3 as experimental endpoints to allow the mice to be sacrificed at multiple timepoints before becoming moribund from the high bacterial burden (~1,500 CFUs). We extracted bacterial genomic DNA from each sample to analyze by Tn-seq as described in Long et al. (2015) and analyzed this data using TRANSIT software (DeJesus et al., 2015), a tool for identifying the frequency of *Himar1* insertions at each site in a genome (Figure 1A).

Comparing the relative abundance of each strain as measured by unique reads of its transposon junction, we identified mutants with increasing, decreasing, and relatively constant population prevalence over time (Supplementary Table S1). Analysis of Day 1 strain abundance showed that all 87 strains were successfully delivered to the lungs in relatively similar numbers (Supplementary Figure S1). Since we were chiefly interested in *PE/PPE* genes that promote virulence, we focused on mutants that decreased in percent abundance with longer *in vivo* incubation. Among these, we identified several genes with known functions or roles relevant to host infection, including *PE19* (Bottai et al., 2012; Ramakrishnan et al., 2015), *PPE51* (Wang et al., 2020), and *PPE68* (Demangel et al., 2004) (Figures 1B–D, blue). Intriguingly, we found that the *PPE71* mutant had similar behavior to these known genes at each timepoint (Figures 1B–D, red). As such, we selected this *PPE71* transposon mutant (henceforth ‘71::Tn’) for subsequent validation using individual strain infections.

The composition of the *PE/PPE* mutant pool shows directional changes over time *in vivo.*

To more comprehensively examine the behavior of the *PE/PPE* mutant pool *in vivo*, we compared the proportion of each individual strain at each timepoint. We detected a strong positive correlation (R^2^ = 0.81) between the abundance of each mutant strain at Week 1 and abundance at Week 3 when normalized to initial abundance at Day 1 (Figure 1E). While the mutant strains showed a range of behaviors between Day 1 and Week 1, including expansion, contraction, or a lack of change, the strains tended to maintain similar behavior between Week 1 and Week 3. For example, we found that strains that decreased in population prevalence at Week 1 tended to decrease further by Week 3 (Figures 1F,G). Relatedly, the overall prevalences of the strains when viewed together was most similar at Week 1 and Week 3 compared to Day 1, which was visibly different (Supplementary Figure S1). Collectively, these patterns suggest a period of rapid change in the composition of the pool between Day 1 and Week 1, followed by a continued elaboration of these same changes in the interval from Week 1 to Week 3. Hence, these changes appear to be directional rather than the result of random noise.

### *PPE71* transposon mutant, complemented, and overexpressor strains show no differences from WT *M.tb in vitro* or in macrophages

**Figure 2:**
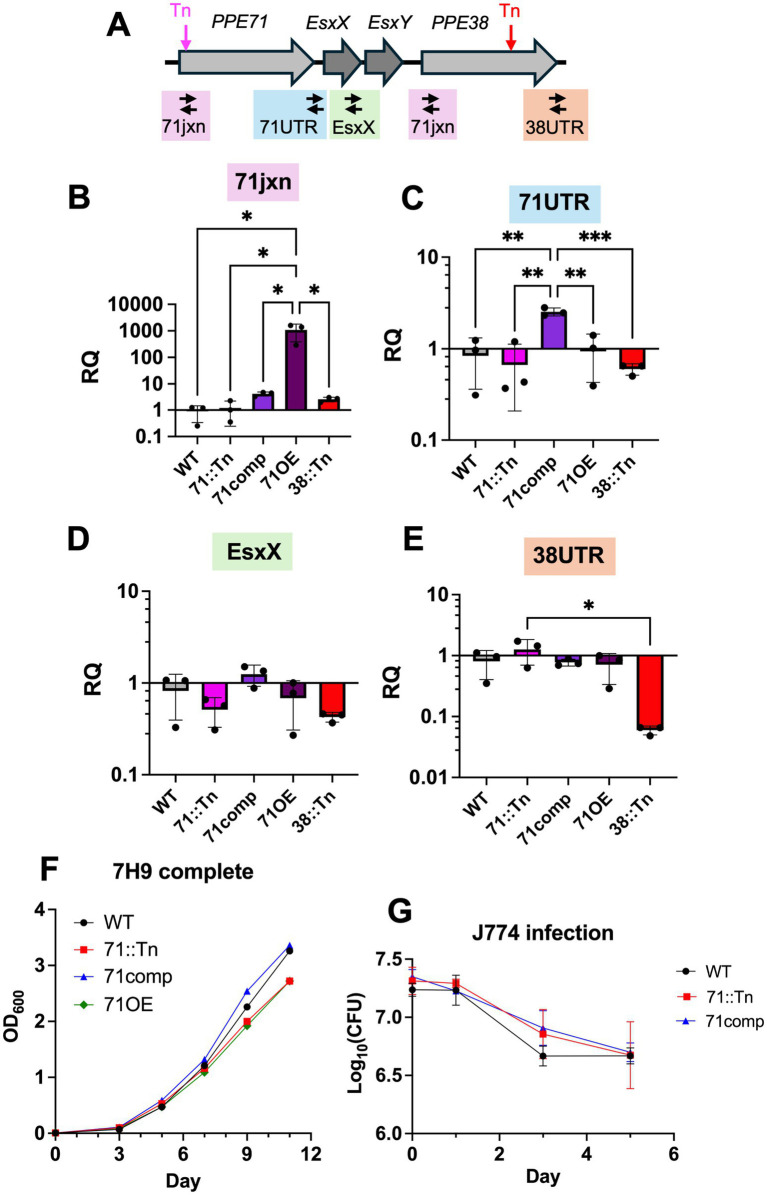
*PPE71* variant strains display no growth differences *in vitro* or in macrophages. **(A)** Schematic of the *PPE71–38* locus, including the location of the transposon insertions in the *PPE71* gene in the 71::Tn strain (pink) and in the *PPE38* gene in the 38::Tn strain (red). Also indicated are the locations of the qRT-PCR primer amplicons, which span the *PPE71* transposon junction (71jxn), the 3′ untranslated region (UTR) of *PPE71* (71UTR), the *esxX-Y* genes (esxX), and the 3’ UTR of *PPE38* (38UTR). Notably, the 71jxn primers bind to the same location in both the *PPE71* and the *PPE38* genes. **(B–E)** Relative transcriptional expression by qRT-PCR of the 71jxn **(B)**, 71UTR **(C)**, EsxX **(D)** and 38UTR **(E)** regions of the *PPE7–38* locus, normalized to the median of the WT strain. Transcript levels were normalized to the expression of 16S rRNA. All significant comparisons by one-way ANOVA are shown. RQ: relative quantification (*n* = 3, *: *p* < 0.05; **: *p* < 0.01). **(F)** Growth curves of *M.tb* WT, 71::Tn, 71comp, and 71OE strains in complete 7H9 broth. OD_600_: optical density at 600 nm. **(G)** *M.tb* WT, 71::Tn, and 71comp strains were used to infect J774 cells at an MOI of 5. Cells were harvested at 0, 1, 3, and 5 days to measure *M.tb* CFUs. (*n* = 3 each).

To further study the *PPE71* transposon strain, we generated a *PPE71* complemented strain (henceforth ‘71comp’) in the 71::Tn background and a *PPE71* overexpression strain (henceforth ‘71OE’) in the WT CDC1551 background. We evaluated the expression of *PPE71* in these strains using a series of qRT-PCR primers spanning the *PPE71* gene locus (Figure 2A). The *PPE71* locus contains a second downstream *PPE* gene, *PPE38*, which is identical to *PPE71* except for an additional 7 amino acids (GGAGAGM) in its highly GC-rich C-terminus (McEvoy et al., 2009). Hence, while we could demonstrate a > 1,000-fold increase in *PPE71* mRNA in the 71OE strain compared to WT, our primer pairs directed against *PPE71* transcripts failed to show a loss of *PPE71* in the 71: Tn strain due to the intact copy of *PPE38* (Figure 2B). However, priming against the unique 3′ untranslated region (UTR) of *PPE71* demonstrated that the 71comp strain successfully re-introduced *PPE71* expression (Figure 2C). The *PPE71* overexpression construct does not include the native 3’ UTR and thus did not show increased amplification with these primers. Interestingly, we did not detect any difference in expression of the *PPE71* 3’ UTR or downstream *esxX* gene in the 71::Tn mutant, suggesting that transcription of the remainder of the *PPE71* locus remained intact despite the insertion of over 1 kb of transposed DNA into the *PPE71* gene (Figures 2C,D). As an internal control, we used the *PPE38* transposon mutant (‘38::Tn’), which behaved similarly to WT except for a prominent decrease in the unique 3’ UTR of *PPE38*, consistent with a transposon insertion in the C-terminus of *PPE38* (Figure 2E). In sum, the *PPE71* complementation and overexpression constructs increased *PPE71* expression as expected.

During cultivation of the 87 *PE/PPE* transposon mutant strains, we seeded each strain at OD_600_ 0.006 into individual cultures and allowed them to grow for 9 days. On the day of infection, the strains ranged OD_600_ between 0.6 and 1.5, suggesting approximately similar growth and agreeing with previous reports about the general non-essentiality of these genes *in vitro* (Sassetti et al., 2003; DeJesus et al., 2017). Analogously, the 71::Tn, 71comp, and 71OE strains each displayed similar growth kinetics to WT *M.tb* in complete 7H9 broth (Figure 2F). Next, we infected WT, 71::Tn, and 71comp strains into the J774 mouse macrophage-like cell line and assessed bacterial CFUs at 0, 1, 3, and 5 days post-infection. We found no differences in CFU counts over time between these strains in this cellular infection model (Figure 2G). Hence, the large decrease in the 71::Tn mutant by Week 1 in the pooled mouse infection was not recapitulated *in vitro* or in a macrophage infection model.

### Behavior of *PE/PPE* mutants in a macrophage infection model is broadly inconsistent with *in vivo* behavior

Initially, we had anticipated that macrophage infection would be a reasonable predictor of *PE/PPE* transposon mutant behavior *in vivo*, as infection of alveolar macrophages is a vital early step in the *M.tb* life cycle (Ramakrishnan, 2012; Flynn et al., 2011). We were thus surprised that the 71::Tn mutant did not demonstrate the same attenuation in a J774 infection model as it had shown *in vivo*. To assess whether this inconsistency was unique to the 71::Tn mutant, we performed a high-throughput infection of 22 *PE/PPE* transposon mutants, including 71::Tn, alongside WT *M.tb* in bone marrow-derived macrophages (BMDMs) from C57BL/6 mice. All 22 transposon mutant strains that we selected had shown decreasing population prevalence over time in the initial pooled infection. However, at Day 2 post-infection in BMDMs, these strains exhibited a diverse range of behaviors compared to their Day 0 counts (Supplementary Figures S2A,B). While some transposon mutants, such as *PE19*, showed a decrease akin to their behavior in the pooled infection, others such as *PPE68* remained constant or grew slightly in the BMDMs. Taken together, there was no correlation between the kinetics of these strains in BMDMs and their behavior *in vivo* at either Week 1 (*R*^2^ = 0.003) or Week 3 (*R*^2^ = 0.029) timepoints in mice (Supplementary Figures S2C,D). This inconsistency suggests that each model may be eliciting different properties of the *M.tb* mutants.

### *M.tb PPE71*::Tn is attenuated in mice

**Figure 3:**
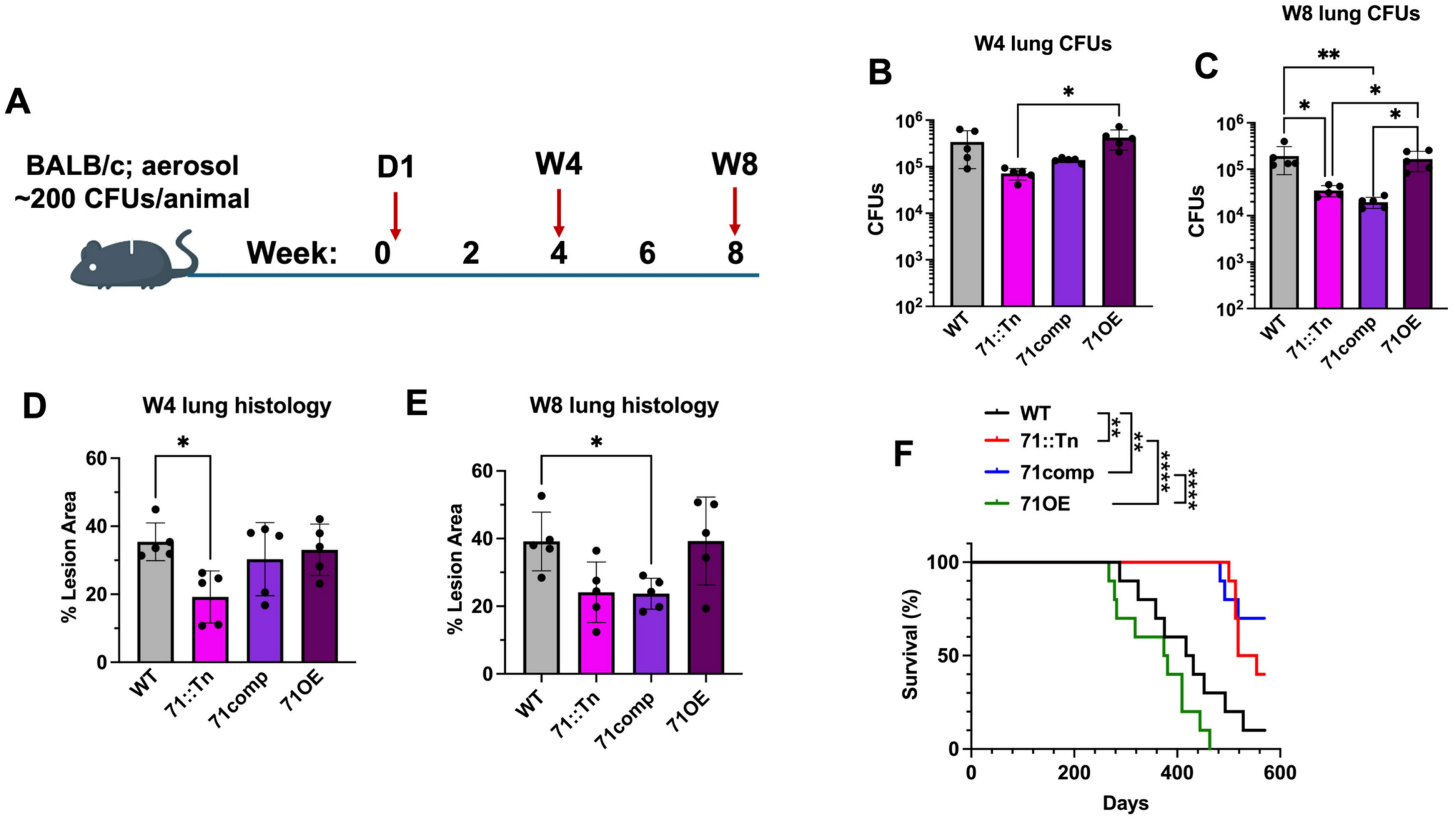
Single strain infections recapitulate attenuation of the PPE71::Tn mutant strain. **(A)** Design for medium-dose single strain aerosol infection of *M.tb* strains into BALB/c mice. Animals were sacrificed on Day 1 to measure inocula, then on Week 4 and Week 8 as experimental timepoints. **(B,C)** Lung CFUs for Week 4 **(B)** and Week 8 **(C)** timepoints. All significant comparisons by one-way ANOVA are shown (*n* = 5, *: *p* < 0.05; **: *p* < 0.01). **(D,E)** Percent of the lung area occupied by lesions at Week 4 **(D)** and Week 8 **(E)** post-infection, as quantified using ImageJ from H&E-stained histology images. All significant comparisons by one-way ANOVA are shown (*n* = 5, *: *p* < 0.05). **(F)** Kaplan–Meier survival curves for BALB/c mice infected with the indicated *M.tb* strains (~200 CFUs/animal; aerosol) (*n* = 9–10 each, **: *p* < 0.01, ****: *p* < 0.0001 by Mantel-Cox log-rank test).

To validate the findings of our *in vivo* screen, we individually infected *M.tb* WT, 71::Tn, 71comp, and 71OE strains into BALB/c mice by aerosol route. We used a dose of around 200 CFUs per animal to allow us to examine the later timepoints of Week 4 and Week 8 (Figure 3A). We found no significant differences in Day 1 lung CFUs for the latter three strains, although the WT *M.tb* group received a moderately higher inoculum (Supplementary Figure S3A). We observed reduced lung CFUs in the 71::Tn strain compared to the WT and 71OE strains, particularly at the Week 8 timepoint, at which 71::Tn exhibited a 5.5-fold decrease compared to WT (Figures 3B,C). The 71comp strain appeared partially attenuated at Week 4 and showed little difference from the 71::Tn strain at Week 8.

To assess lung immunopathology, we subjected fixed lungs to staining with hematoxylin and eosin (H&E) and quantified the percent area of each lung occupied by inflammatory lesions. Using this method, we found significantly reduced inflammation in the 71::Tn strain group at Week 4, though this trend had lessened to non-significance by Week 8 (Figures 3D,E). Instead, at Week 8, the 71comp strain group had significantly lower inflammation than the WT, suggesting that complementation did not restore the transposon mutant strain to WT-like virulence (Figure 3E). Overall, the lungs from mice infected with the 71::Tn and 71comp strains appeared to have less inflammation than the lungs from mice infected with 71OE or WT (Supplementary Figure S4). We did not find any difference between the 71OE and WT strains in terms of lung lesion area at either the Week 4 or Week 8 timepoints.

As part of this same infection study, we reserved 10 animals from each group for a survival study. We observed no statistical difference in survival between the WT (median survival 417 days) and 71OE (median survival 374 days) groups. Mice from the 71::Tn (median survival 518 days) and the 71comp (median survival >570 days) survived for significantly longer than the WT group (Figure 3F). Indeed, 7 out of 10 mice in the WT group and all 10 mice in the 71OE group reached survival endpoints before a single animal in either of the 71::Tn and 71comp groups. A similar effect was observable in the animal body weights, which began to decline around week 25 (day 175) in the WT and 71OE groups but remained constant in the 71: Tn and 71comp strains through week 38 (day 266), at which point the first animal reached an endpoint and body weight tracking was discontinued (Supplementary Figure S3B). Hence, the 71: Tn strain was attenuated in bacterial burden, pathology, and mortality studies.

## Discussion

The *PE/PPE* genes of *M.tb* remain understudied even though several members are known to promote *M.tb* virulence. Here, we adapted a Tn-seq design for use *in vivo* to identify *PE/PPE* genes that are required for *M.tb* growth during lung infection. We observed consistent changes in the composition of our 87-strain pool between animal replicates and across successive timepoints, suggesting that we were able to reliably elucidate the differential virulence of these strains. We selected the *PPE71* transposon mutant strain, which decreased in prevalence in the pooled experiment, for further study using an individual mutant infection design. Mice infected with the 71::Tn strain had reduced lung bacterial burden, decreased lung inflammation, and a longer median survival than mice infected with WT *M.tb*. The behavior of the 71::Tn strain in individual infection was consistent with its decrease in population prevalence in the pooled infection. Thus, our *in vivo* Tn-seq screen successfully predicted the attenuation of an uncharacterized *M.tb* transposon mutant.

Pooled studies of genetically diverse pathogens come with the inherent challenge of minimizing founder effects, in which a small non-representative subpopulation may overwhelm the remaining diversity of the pool (Meyr, 1942). For this reason, we opted to individually grow each transposon mutant strain and pool these fresh cultures immediately prior to infection rather than either growing the pooled strains together or infecting from frozen stocks, each of which may introduce bias. Additionally, we chose to infect with a high CFU dose to maximize the number of bacteria representing each individual strain. From our prior experience, 1,500 CFUs (15–20 bacteria per mutant strain) was the approximate maximum dose that could be reproducibly achieved with our nebulization setup. Of note, the mouse lung contains on the order of 10^6^ alveoli (Knust et al., 2009), so the chance that any two bacilli of different strains would co-infect the same compartment was minimal. Thus, we reasoned that direct cross-complementation of different mutants was unlikely. We were able to detect each strain in each animal across all three timepoints, suggesting that the initial diversity of the pool was maintained throughout the experiment. Also, we observed consistent behavior of the strains across the animal replicates for any given timepoint, which gave us confidence that the changes we observed were meaningful rather than stochastic. While we have mainly focused on mutant strains with decreasing population prevalence, we note as well that several mutants increased in prevalence over time. In particular, a high proportion of *PE_PGRS* family gene mutants fall into this category. It is possible that mutations in these genes have a less attenuating effect or even a virulence promoting effect compared to mutations in other *PE/PPE* genes. Given the high frequency of mutations in select *PE_PGRS* family genes among *M.tb* clinical isolates (Copin et al., 2014; Talarico et al., 2005; Talarico et al., 2008), this property may merit further investigation.

To supplement our *in vivo* screen, we individually infected a subset of 22 *PE/PPE* gene mutants into mouse BMDMs with the goal of rapidly identifying promising candidate mutants for further study. Surprisingly, we observed no correlation between the *in vivo* results and the behavior in this macrophage infection. Indeed, several of the mutants with the most dramatic decreases during animal infection, such as *PPE51* and *PPE68*, demonstrated a slight growth in macrophages. This discrepancy may be explained by the comparative simplicity of the macrophage system, which lacks the other cell types and surrounding tissue architecture found in the lung (McCaffrey et al., 2022). The macrophage environment may thereby permit a greater range of *M.tb* genomic mutations than would be tolerable in the more stringent host environment. Additionally, this lack of correlation between the two experiments may reflect intrinsic differences in *M.tb* control by macrophage lineage, as different populations of mouse macrophages are known to display disparate *M.tb* control (Huang et al., 2018), and *ex vivo* BMDMs show underlying metabolic differences compared to alveolar macrophages (Vigeland et al., 2025). We acknowledge the use of two different macrophage types, mouse BMDMs and J774 cells, respectively used in our 22-mutant screen and in our *PPE71* validation experiments, though we believe these cell types provide similar results. Notably, the culture conditions of the *M.tb* mutant strains prior to infection and following macrophage lysis or lung homogenization were identical across both experiments, so we do not believe that the differential behavior reflects differences in *in vitro* growth. Overall, the *in vivo* findings likely better represent the true effects of these mutations on pathogenesis, as mutants in *PPE51* (Wang et al., 2020) and especially *PPE68* (Demangel et al., 2004) would be expected to be attenuated based on the functions of these proteins in nutrient uptake and phagosome disruption, respectively.

While analyzing the Tn-seq data, we noted irregularities in the several of the transposon mutant strains. Principally, 19 of the 87 strains (22%) had their transposon insertions in genomic regions other than *PE/PPE* genes, as annotated by TRANSIT software (DeJesus et al., 2015). Based on our analysis, we believe that each of these strains has a single transposon insertion but that this insertion is not in the *PE/PPE* gene under which the mutant was catalogued. These strains were included in the initial analysis, although we did not pursue any of them for further study, as we were mainly interested in *PE/PPE* genes. Since these are true transposon mutants, their phenotypes may still be of interest in studies of the relevant genes. In several of these cases, we were able to identify partial matches that resembled *PE/PPE* gene sequences; however, when we manually examined our raw sequencing data, we consistently agreed with the non-*PE/PPE* annotations output by TRANSIT. We have detailed these ‘partial matches’ in Supplementary Table S1. All partial matches were to PE_PGRS or PPE-MPTR proteins, two subfamilies that contain large domains of repetitive sequences (Cole et al., 1998; Gey van Pittius et al., 2006). These genomic repeats are challenging to sequence and differentiate from one another (Ates, 2020), which may explain why these strains appear to have been historically mis-annotated.

We found that restoring *PPE71* under its native promoter was only partially able to complement the 71::Tn mutant strain. While it is possible that this reflects a polar effect (i.e., transposon insertion into *PPE71* may have disrupted the downstream genes in the locus) or a consequence of incomplete gene deletion, we found that the remaining genes in the *PPE71* locus were transcribed at WT levels. Another possibility is the presence of one or more bystander mutations in the 71::Tn strain that could be driving attenuation. Disruption of the *PPE71* locus in *M.tb* has previously been shown to cause hypervirulence (Ates et al., 2018a; Koleske et al., 2025), the opposite phenomenon to what we observe with this transposon mutant strain. Hence, we cannot rule out a *PPE71*-independent mechanism to explain the attenuation of the 71::Tn strain. For addition information regarding the growth of *PPE71* variant strains *in vitro*, we direct the reader to the excellent prior work examining this gene locus in *M.tb* (Ates et al., 2018a), which has found no *in vitro* growth defect of a *PPE71* deletion strain through Day 28. We have also examined an unmarked deletion of the *PPE71* locus using similar complementation and overexpression constructs in other work (Koleske et al., 2025), in which we observed no growth defect of *PPE71* deletion or complement strains *in vitro* and a slight growth defect of the *PPE71* overexpressor strain at Day 9. Indeed, the risk of polar effects influenced our choice of candidate for further study: we considered only *PE/PPE* genes that are co-operonic with exclusively other ESX-5 substrates or comprise single-gene operons. This most notably caused us to exclude *PPE1*, which is the first gene in a six-gene operon involved in metallophore synthesis, due to the possibility that polar loss of the downstream enzymes would represent the true source of that mutant’s strongly attenuated phenotype (Mehdiratta et al., 2022).

In sum, we present a pooled screen of *M.tb PE/PPE* gene transposon mutants infected by aerosol route into mouse lungs. We find that 27 of 87 (31%) mutants were defective for growth in mouse lungs (Day 1 vs. Week 3). Previous studies using high-density *M.tb* transposon libraries have shown that around 600–700 of the 3,924 protein-coding genes (15–18%) are required for survival in mice (Sassetti et al., 2003; DeJesus et al., 2017). Thus, our findings indicate that the PE/PPE protein family may be enriched in virulence genes relative to the whole *M.tb* genome. We also note the inability of an *in vitro* model or a macrophage infection model to properly identify *PE/PPE* gene mutants that are attenuated in mice. Hence, we demonstrate the advantages of conducting a screen for *M.tb* virulence factor mutants *in vivo*, which can be facilitated using marked mutants such as transposon insertion strains. We find high variability in the behavior of different *PE/PPE* gene mutants *in vivo*, indicating that the diverse members of these families likely exhibit a range of different contributions to *M.tb* virulence. Future studies of PE/PPE proteins may prioritize candidates based on the *in vivo* behavior we find here.

## Data Availability

The datasets presented in this study can be found in online repositories. The names of the repository/repositories and accession number(s) can be found here: https://www.ncbi.nlm.nih.gov/, PRJNA1256389.
