# Supplementary material for: In vivo profiling of the PE/PPE proteins of Mycobacterium tuberculosis reveals diverse contributions to virulence
Source: Front Microbiol. 2025 Jun 18;16:1634229. doi: 10.3389/fmicb.2025.1634229 (PMC12213770; doi:10.3389/fmicb.2025.1634229)
Supplement: Supplementary file 2 [file Data_Sheet_1.pdf]

Supplementary Material for:

In vivo profiling of the PE/PPE proteins of *Mycobacterium tuberculosis* reveals diverse contributions to virulence

Benjamin Koleske[1], Jessica Shen[1], Manish Gupta[1] & William R. Bishai[1*]

[1]Center for Tuberculosis Research, Department of Medicine, Johns Hopkins University School of Medicine, Baltimore, MD 21287, USA

***Correspondence:**
William R. Bishai
1550 Orleans Street
CRBII, Room 108
Baltimore, MD 21231-1001
Telephone: 410-955-3507
Email: wbishai1@jhmi.edu

Supplementary Figures

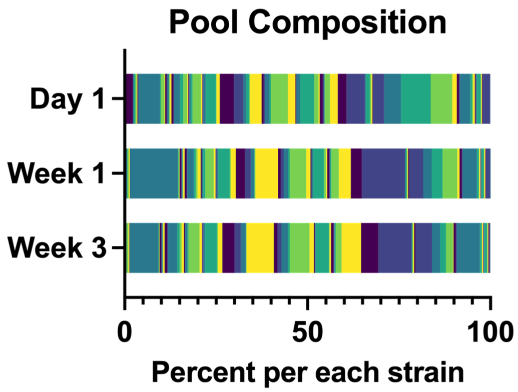

Figure S1: Composition of the *PE/PPE* mutant pool over time *in vivo*.
Each colored rectangle represents a single transposon mutant strain. The width of each rectangle represents the mean percent of the total pool occupied by each strain across 9–10 animal replicates. The overall composition of the pool appears more similar between the Week 1 and Week 3 timepoints compared to the Day 1 timepoint, suggesting a period of rapid initial change between Day 1 and Week 1.

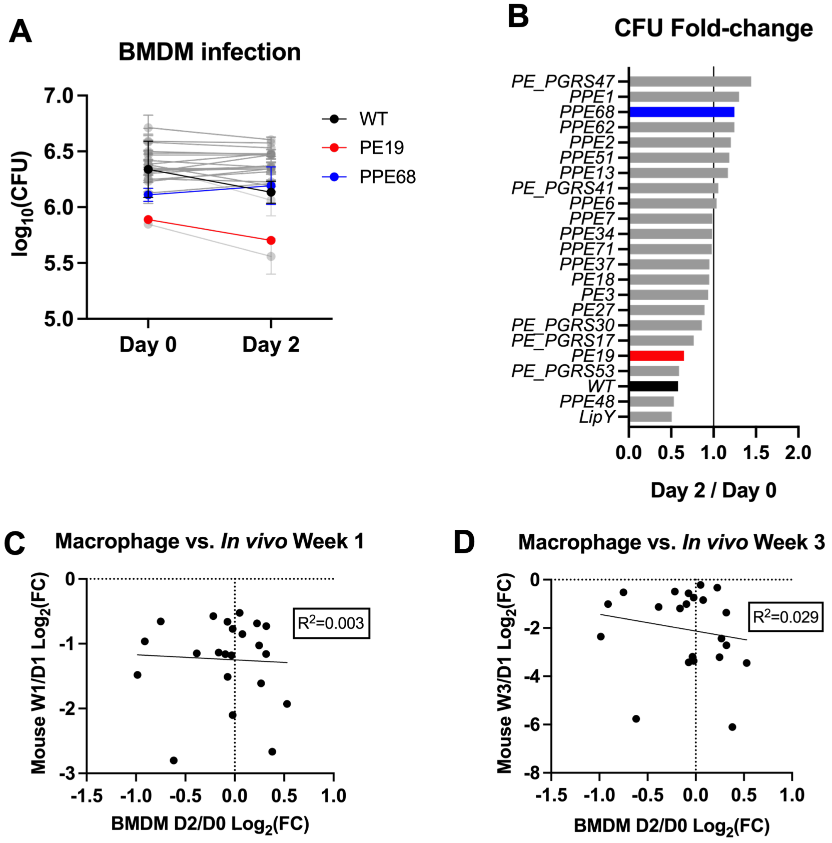

Figure S2: Profiling 22 *PE/PPE* transposon mutant strains in a mouse BMDM infection model.

(A) *M.tb* WT and 22 transposon mutant strains were used to infect C57BL/6 bone marrow-derived macrophages (BMDMs) at an MOI of 2. Cells were harvested after 0 or 2 days to measure *M.tb* CFUs. *M.tb* WT is indicated in black, the *PE19* mutant in red, and the *PPE68* mutant in blue. (n=2 per strain per timepoint.)

(B) Mean fold-change in *M.tb* CFUs from Day 0 to Day 2 for each strain tested by BMDM infection. The vertical line at 1.0 indicates no change. (n=2 per strain per timepoint.)

(C–D) Correlation plots comparing the change in BMDM CFUs (from Day 0 to Day 2, as $\log_2$(fold-change)) for each mutant compared to the changes in the *in vivo* pooled experiment. Change in BMDM CFUs is compared to *in vivo* CFUs for Week 1 (C) or Week 3 (D), each normalized to Day 1 by $\log_2$(fold-change). Behavior in mouse macrophages shows no correlation with behavior *in vivo* at either timepoint.

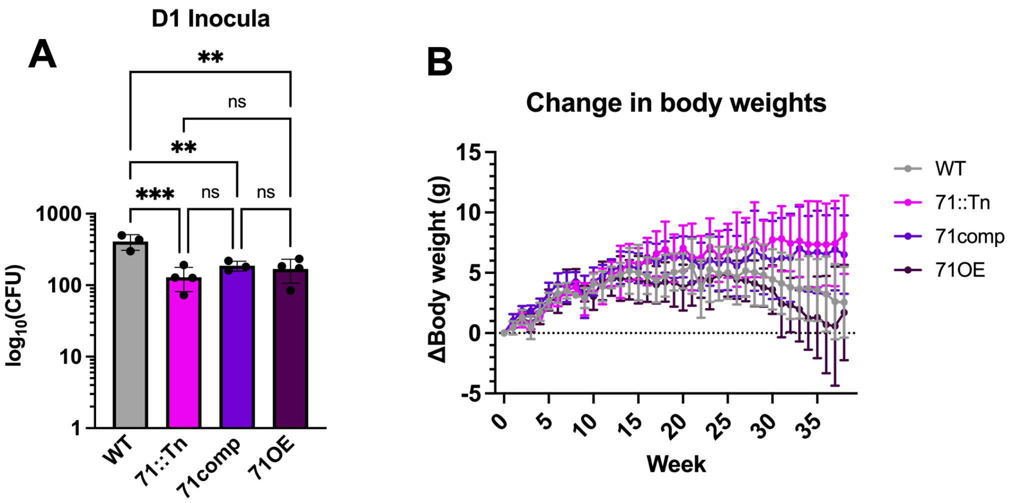

Figure S3: Day 1 inocula and longitudinal body weights for mouse infection with individual *M.tb PPE71* strains.

(A) Total lung CFUs at Day 1 post-infection were counted to obtain inocula. WT *M.tb* had a significantly higher inoculum than the 71::Tn, 71comp, and 71OE strains, which were otherwise not significantly different from one another by one-way ANOVA. (n=3-4, **: $p<0.01$, ***: $p<0.001$.)

(B) Change in body weight (compared to starting body weight at Week 0) for mice infected with WT *M.tb* and *PPE71* variant strains through Week 38 of infection, at which time body weight measurements were discontinued due to the occurrence of the first endpoint. (n=5 each.)

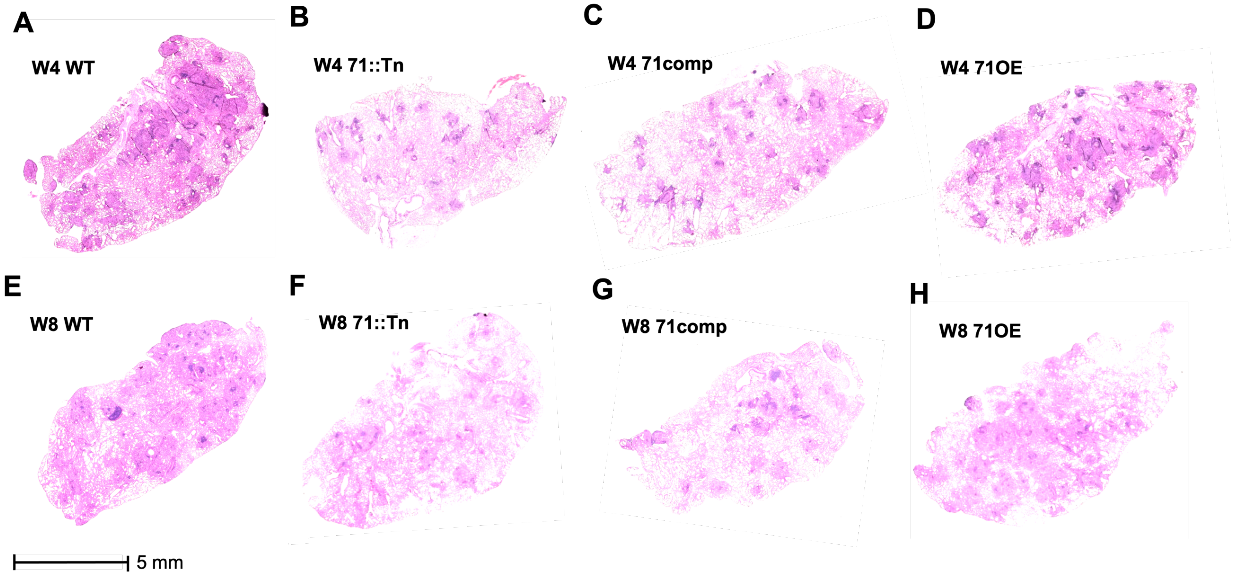

Figure S4: Representative mouse lung histology images.
(A–D) Representative Week 4 H&E histology images for mice lungs infected with *M.tb* WT (A) 71::Tn (B), 71comp (C), and 71OE (D) strains.
(E–H) Representative Week 8 H&E histology images for mice lungs infected with *M.tb* WT (E) 71::Tn (F), 71comp (G), and 71OE (H) strains.

Supplementary Tables

Table S1. Abundance of each transposon mutant in each mouse by Tn-seq.
Locations and relative abundances of *Himar1* transposons identified in the genomic DNA obtained from cultured mouse lung homogenate, assessed by TRANSIT software. Counts for each TA site insertion are normalized to total counts for the respective animal. Gene assignments are provided. For sites that did not map to a *PE/PPE* gene, best partial matches to a *PE/PPE* gene are provided where possible. Strains that were subsequently tested in bone marrow-derived macrophages are indicated. (Animals 1–10: Day 1; animals 11–20: Week 1; animals 21–30: Week 3.)

(N.B.: Table S1 is provided as a separate Microsoft Excel file due to its large size.)

**Table S2. Plasmids used in this study.**		
**Plasmid**	**Description**	**Reference**
pMH94-hyg	L5 *int*, *M.smegmatis attP*, *ampR* (*E.coli*), hyg*R* (*M.tb*)	(Lee et al. 1991)
pMH94-hyg-PPE71	pMH94-hyg + [422nt upstream of *PPE71* to 111nt downstream of *PPE71*]	This work
pSD5	*OriM*, *M.leprae hsp65* promoter, *ampR* (*E.coli*), *kanR* (*M.tb*)	(DasGupta et al. 1998)
pSD5-71OE	pSD5 + [60nt upstream of *PPE71* to 28nt downstream of *PPE38*]	This work

Table S3. Oligonucleotides used in this study.

Oligonucleotide	Sequence (5'–3')
PPE71_24f	GAATTCGAGCTCGGTACCCGGGGATCCTCTAGATTCCGTTCGGTAGTGCGAT
PPE71_24r	GCAGAGATGGTGCCCTTGGTGGTCGACTCTAGTCATAAACCGAGTAGCCACCA
PPE71_pSD5_f	GCGATATCCGGAGGAATCACTTCCATGTTTGTGTCTGGAGAGTGGTAGG
PPE71_pSD5_r	CCATTGAAGACCGGGCCAGAACGCGGCAAAGACCCCGACCAATC
pMH94_Fseq	GAAAATACCGCATCAGG
pMH94_Rseq	GAATAGACCGGGACAAGG
pSD5_insert_FWD	AGCGTAAGTAATGGGGGTTGTCG
pSD5_insert_REV	ATATATTCGGTCGCTGAGGCTTG
16S_q_F	GCCGTAAACGGTGGGTACTA
16S_q_R	TGCATGTCAAACCCAGGTAA
PPE71_q_T1F	ATCACCGCATCAAACGAGGA
PPE71_q_T1R	TGGATTTTTCGTGGTTGCCG
PPE71UTR_qF	TTGCGTTGATGACATTGCGG
PPE71UTR_qR	AAACCGAGTAGCCACCACAC
EsxX_qF	CGCAAATCCGCATGTGGTG
EsxX_qR	CCAAGGCCAGGGATTTCCAA
PPE38UTR_qF	GGTCCGTGACGCGAATAACT
PPE38UTR_qR	TCATCGCCAATGCCCTTTCT
Adapter_constant	TACCACGACCA-*NH2*
Adapter_variable	ATGATGGCCGGTGGATTTGTG*NN*A*NN*A*NNN*TGGTCGTGGTAT
Short_adapter	ATGATGGCCGGTGGATTTGTG
Short_transposon	TAATACGACTCACTATAGGGTCTAGAG
HN_transposon_1	AATGATACGGCGACCACCGAGATCTACACTCTTTCCCTACACGACGCTCTTCCGATCTCGGGGACTTATCAGCCAACC
HN_transposon_2	AATGATACGGCGACCACCGAGATCTACACTCTTTCCCTACACGACGCTCTTCCGATCTTCGGGGACTTATCAGCCACCC
HN_transposon_3	AATGATACGGCGACCACCGAGATCTACACTCTTTCCCTACACGACGCTCTTCCGATCTGATACGGGGACTTATCAGCCACCC
HN_transposon_4	AATGATACGGCGACCACCGAGATCTACACTCTTTCCCTACACGACGCTCTTCCGATCTATCTACGGGGACTTATCAGCCAACC
HN_barcode_XX-1*	CCAGCAGAAGACGGCATACGAGATXXXXXXXXGTGACTGGAGTTCAGACGTGTGCTCTTCCGATCTGTCAATGATGGCCGGTGGATTTGTG
HN_barcode_XX-2*	CCAGCAGAAGACGGCATACGAGATXXXXXXXXGTGACTGGAGTTCAGACGTGTGCTCTTCCGATCTCGTCCATGATGGCCGGTGGATTTGTG
HN_barcode_XX-3*	CCAGCAGAAGACGGCATACGAGATXXXXXXXXGTGACTGGAGTTCAGACGTGTGCTCTTCCGATCTACAGTCCCATGATGGCCGGTGGATTTGTG
HN_barcode_XX-4*	CCAGCAGAAGACGGCATACGAGATXXXXXXXXGTGACTGGAGTTCAGACGTGTGCTCTTCCGATCTTAGTGGATGATGGCCGGTGGATTTGTG

**Table S4. Barcode sequences used for each Tn-seq library.**	
**Oligonucleotide**	**Sequence (5'–3')**
Barcode_01	TGTTCCGA
Barcode_02	TTCCGGAG
Barcode_03	GCCGATGT
Barcode_04	CATGATCG
Barcode_05	CGCGCGGT
Barcode_06	ACACGATC
Barcode_07	AAGTAGAG
Barcode_08	GAGATCTT
Barcode_09	AACCTGGA
Barcode_10	CTAGACGA
Barcode_11	AGTGGTGA
Barcode_12	GACCTACA
Barcode_13	TACGTCCA
Barcode_14	CGGTGATA
Barcode_15	AAGCGCTA
Barcode_16	GTAACCAG
Barcode_17	TATCGAGG
Barcode_18	TGTCATGG
Barcode_19	TGATGACG
Barcode_20	TCTATGCG
Barcode_21	TTCTCTCG
Barcode_22	TGACTGTG
Barcode_23	AGCGATTG
Barcode_24	TGCCATAC
Barcode_25	ACTTAGCC
Barcode_26	CGTATTCC
Barcode_27	CCATTCTC
Barcode_28	TACACCGT
Barcode_29	GCCTATCT
Barcode_30	TCCGACTT
